# Active thermal-fuse for stopping blue light leakage of white light-emitting diodes driven by constant current

**DOI:** 10.1038/s41598-022-16587-4

**Published:** 2022-07-20

**Authors:** Ching-Cherng Sun, Quang-Khoi Nguyen, Tsung-Xian Lee, Shih-Kang Lin, Chi-Shou Wu, Tsung-Hsun Yang, Yeh-Wei Yu

**Affiliations:** 1 Department of Optics and Photonics, National Central University, ChungLi, 32001 Taiwan; 2 Department of Electrophysics, National Yang Ming Chiao Tung University, HsinChu, 30010 Taiwan; 3 Graduate Institute of Color & Illumination Technology, National Taiwan University of Science and Technology, Taipei, 10607 Taiwan

**Keywords:** Lasers, LEDs and light sources, Other photonics

## Abstract

In this study, we proposed and demonstrated a circuit design for solving problems related to blue light leakage (e.g., eye damage) when phosphor-converted white light-emitting diodes (pcW-LEDs) overheat. This circuit only needs a positive thermal coefficient thermistor, resistor, and diodes in series and parallel; thus, it can easily be integrated into components. Simulations and corresponding experimental results show that this method can accurately suppress the overheating component’s injection current and allow for LEDs to work normally after returning to the operating temperature. It thus allows the user's eyes to be actively protected, e.g., to avoid exposure to the bluish light when overheating occurs. In addition, the quenching of luminous flux is a signal to remind the user to replace the LED. The proposed method is low-cost, effective, simple, and useful for increasing the quality of LED lighting and biological safety.

## Introduction

**Figure 1:**
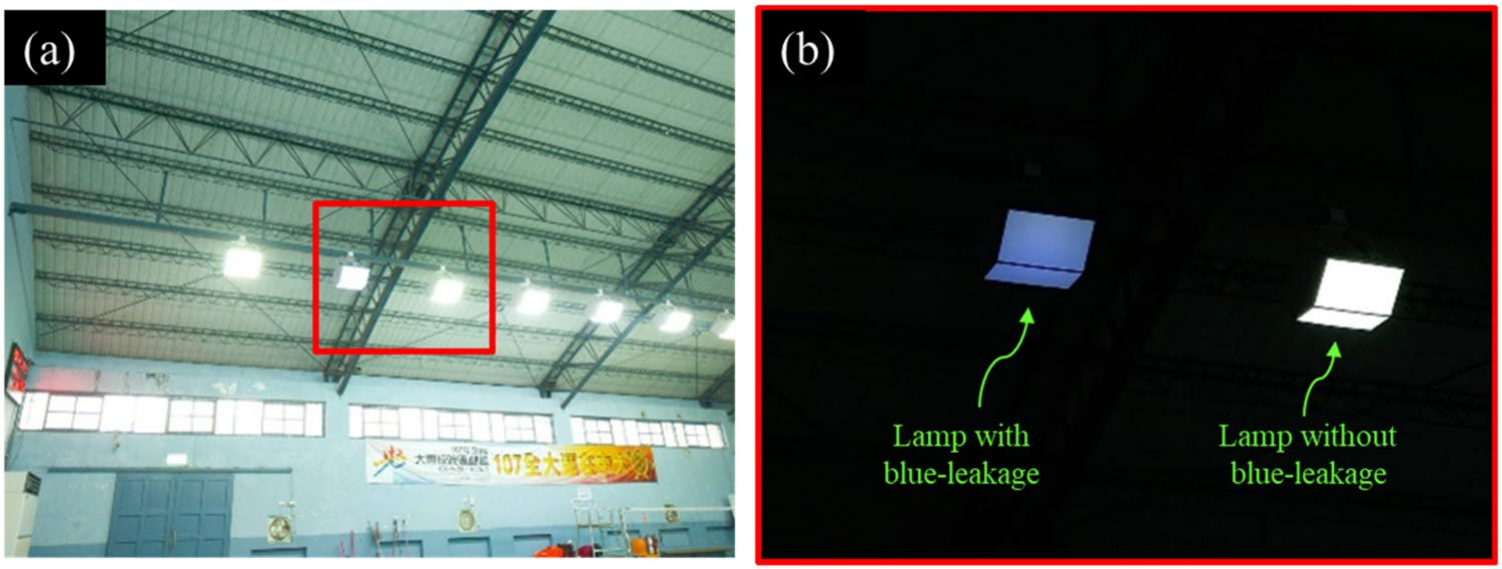
(**a**) Blue leakage problem of phosphor-converted white light-emitting diodes (pcW-LEDs) cannot be seen with the naked eye, and (**b**) can be clearly observed by adjusting the exposure mode of the camera.

**Figure 2:**
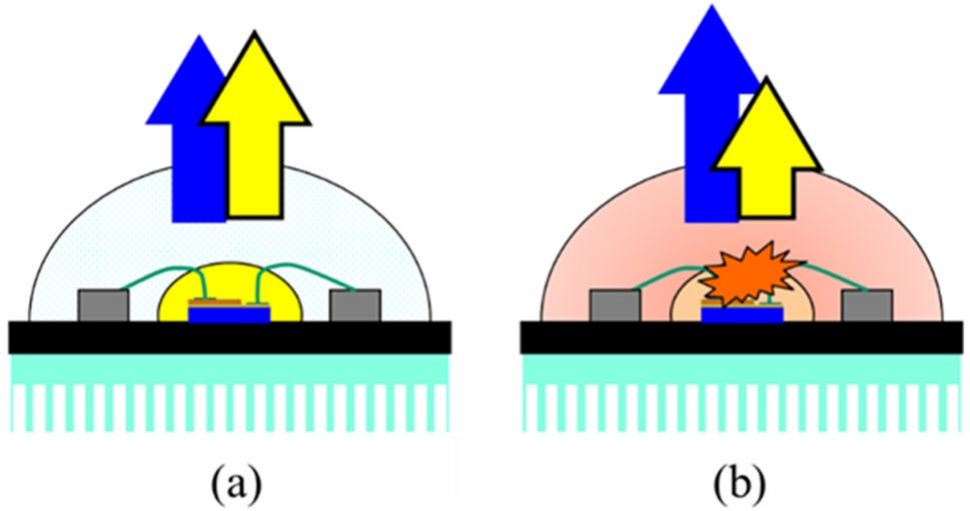
Illustration of heat characteristics and blue to yellow light (B/Y) ratio at (**a**) normal condition, and (**b**) overheat condition.

Solid-state lighting (SSL) has been gradually replacing incandescent light bulbs owing to its advantages including high energy efficiency, a fast response, an acceptable color rendering, a long lifetime, and low cost^1–6^. The white light can be created in different ways, such as via di-chromatic, tri-chromatic, and tetra-chromatic approaches^2^. Among them, the dichromatic approach is widely used owing to its simplicity and efficiency; in this approach, the white light is created by a combination of a blue light-emitting diode (LED) die and yellow phosphor^2^. Such a white light source is commonly referred to as phosphor-converted white light-emitting diodes (pcW-LEDs). In normal conditions, there are two main sources significantly contributing to the heat generation in the operating process of the pcW-LEDs structure: the efficacy of the blue LED die and the conversion efficiency of the phosphor (including its own quantum efficiency and stokes loss). The first heat source is related to the conversion efficiency of the injected electron to the emitted blue photon in the electrical flow through the blue LED dies. The conversion efficiency from electrical to optical power can be higher than 70%; thus, at least 30% of the input electrical power can be transformed to heat^7,8^. The second heat source is the phosphor region, and is related to the Stokes' loss, i.e., the wavelength difference between the excitation and re-emitted wavelengths^2,9^. If the peaks of the blue excitation and yellow emission wavelengths are 450 nm and 550 nm, respectively, the wavelength conversion efficiency (the ratio of excitation wavelength to re-emission wavelength) is approximately 82%. Therefore, approximately 18% of energy from this process is converted to heat. Notably, if normal conditions are not well-maintained, a greater amount of heat will be generated, owing to the dominance of the nonradiative conversion in the blue LED die and phosphor region. It is well-known that heat is an unavoidable problem in pcW-LEDs leading to many negative effects on the quality of the output white light, such as correlated color temperature (CCT) increases, color shifts, efficacy reductions, and degradations of mechanical properties^10–19^. A relatively serious problem related to the heat effect is the phenomenon in which bluish light can be observed even if the lamp is still bright, as shown in Fig. 1. Owing to the thermal decay rate of yellow light being faster than that of blue light, the power ratio of blue to yellow light (B/Y ratio) increases significantly, causing the color of the output white light to become more bluish (corresponding to a very high CCT value, e.g., higher than 10,000 K)^9^. Although it is easy to detect bluish light using an optical instrument, it is not easy to perceive with human eyes. Therefore, once blue light leakage occurs, the user's eyes may be exposed to bluish light without any warning signal. Figure 2 illustrates characteristics of the temperature at normal and abnormal conditions, as well as the effect of overheating on the B/Y ratio. When overheating occurs, the temperatures of the pcW-LEDs become much higher than in normal conditions^24,25^. Moreover, owing to the difference in the thermal decay rate, the B/Y ratios in conditions where overheating occurs are higher than in normal conditions. High temperature in the package volume of a pcW-LEDs could cause thermal quenching of phosphor, resulting in color drift which induces blue-light leakage. Once the phosphor’s temperature increases, the thermal quenching of the phosphor particle reduces the external quantum efficiency such that less yellow light is emitted and more blue light goes through the phosphor volume. If the thermal management is not well enough, the balance between the blue light and yellow light under normal operation will no longer be kept. As a result, it will induce heavy CCT drift or even blue light leakage in the worst condition^26,27^.

Several studies have considered the thermal management of pcW-LEDs^20–26^. Zhang et al. reported a novel blue phosphor of SrLu_2_O_4_: Ce^3+^ prepared by a solid-state reaction; it provided high thermal stability, with 86% of its room-temperature emission intensity remaining at 150 °C^20^. Wang et al. reported the high thermal stability of pcW-LEDs employing Ce:YAG-doped glass^21^. Tang et al. proposed a method for reducing the chromaticity shifts of LEDs using gradient-alloyed Cd_x_Zn_1−x_Se_y_S_1−y_ @ZnS core–shell quantum dots with enhanced high-temperature photoluminescence^22^. Zhao et al. reported using Li substituent tuning of LED phosphors for enhanced efficiency, tunable photoluminescence, and improved thermal stability^23^. Yang et al. proposed a method for stabilizing the CCT in pcW-LEDs based on self-compensation between the excitation efficiency and conversion efficiency of the phosphors^25^. In another report, Yang et al. proposed a practical approach for measuring phosphor temperatures in operating pcW-LEDs; it used a non-contact and instant detection method to remotely monitor emission spectra^26^. Fan et al. reported a packaging technology for high-power white LEDs with a thermal-isolated phosphor coating layer. The technology showed a much higher saturation point for the output luminous flux and a better color characteristic stability under high-power operation conditions relative to conventional LEDs^28^. In general, the solutions in the literature have mainly been focused on improving the thermal stabilization of the phosphor material and packaging technologies. There is still no ideal method for completely eliminating the negative effects of the overheating occurring during the operation processes of pcW-LEDs.

In recent years, reports on blue light hazards have indicated that overexposure to a high amount of blue light and/or exposure over a long time with a low amount of blue light will cause irreversible damage to the retinal tissue in human eyes^29–32^. This risk becomes more dangerous if the user is a child. Thus, the need for a solution to prevent or solve the risk of this blue light hazard has become more urgent than ever, particularly in the process of upgrading the quality of SSLs based on LEDs for a better quality of life. In addition, overexposure to bluish light is a hidden risk (in terms of its biological safety for human eyes) as well as uncomfortable in terms of visualization; notably, it changes the circadian period, thereby affecting human sleep. Accordingly, studies have been conducted to reduce the negative effects of blue light^33–36^.

A lamp showing blue light leakage should be stopped from working to protect human eyes from exposure to bluish light. To the best of our knowledge, there is no study on preventing the blue light leakage from pcW-LEDs when overheating occurs. In this study, we proposed and demonstrated a solution for preventing the blue leakage problem for pcW-LEDs. A circuit was designed to detect overheating and to correspondingly reduce the injected current for pcW-LEDs. As a result, the output white light was significantly suppressed, causing the lamp to dim. The user's eyes were therefore actively protected to avoid exposure to bluish light as overheating occurred. Furthermore, the dimming state of the lamp with the blue light leakage reminded the user to replace it with a new one.

## Blue light leakage by thermal effects

**Figure 3:**
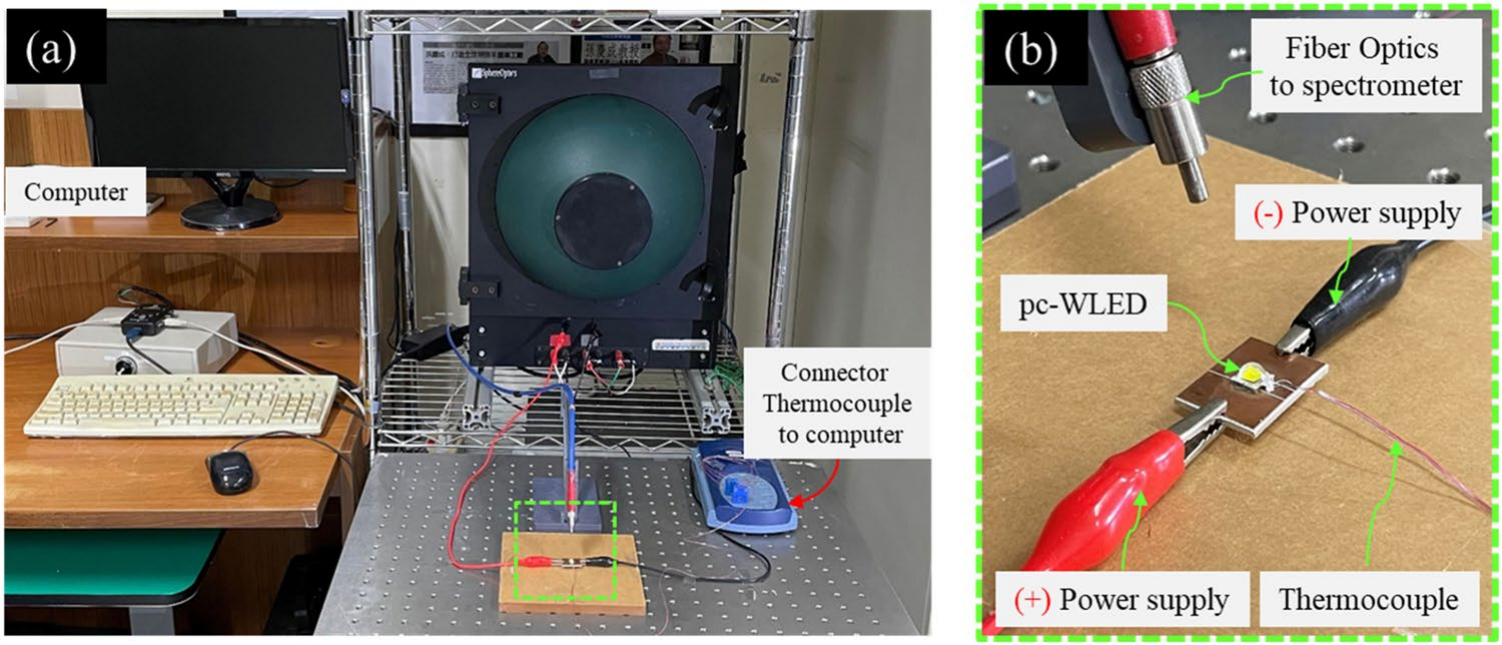
(**a**) Experiment setup for testing the effect of overheating. (**b**) Enlarged part of green dash rectangle in photo (**a**).

**Figure 4:**
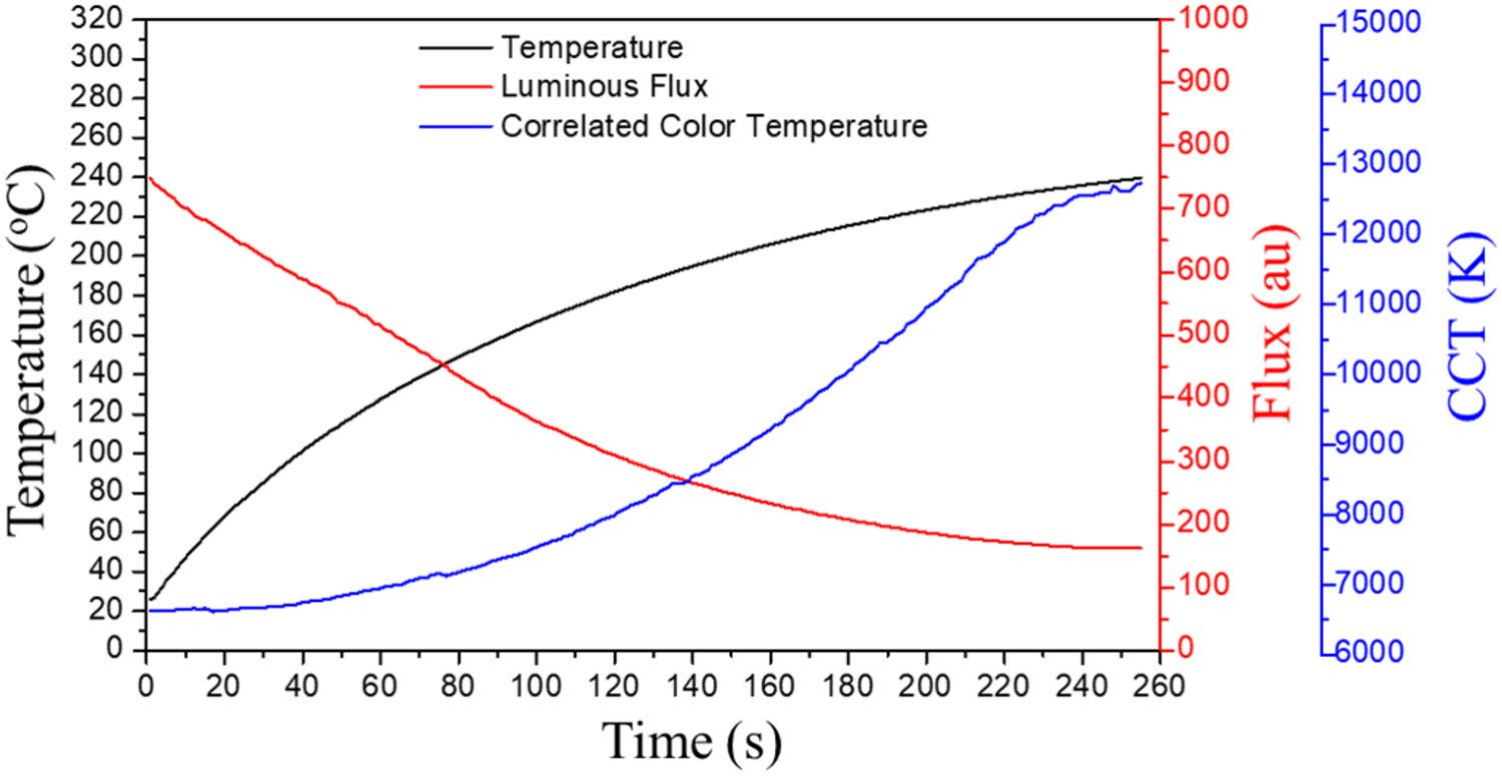
Relation between the optical properties and overheat condition.

In this study, to provide an overview of the characteristics of a lamp with blue light, a condition of overheating was generated in pcW-LEDs. An electrical current driven at 2.5 A and reduced heat dissipation were applied to Cree XML-type pcW-LEDs. The temperature and optical properties were measured by a thermocouple type T and spectrometer, respectively. The corresponding experiment setup and its results are shown in Figs. 3 and 4, respectively. The results shown in Fig. 4 indicate that when overheating occurs in the pcW-LEDs, the effect on the color performance of the output white light is serious, even if the lamp remains bright. The CCT increases from 6500 K up to a very high value (e.g. higher than 8000 K, and reaching up to 12,000 K) corresponding to the bluish color. The temperature shows values in a very high-temperature range, i.e., 180 °C to 240 °C.

In general, the relationship between blue leakage phenomena and temperature can clearly be observed from the results for the CCT and temperature. As the output white light becomes bluish light, the lamp should not be used anymore to avoid damage to human eyes, and the current lamp should be replaced with a new one. Thus, finding a way to halt the operation of the pcW-LEDs when a blue light leakage appears is one option for preventing the negative effects of blue light on users in an illuminated environment.

## Circuit Design

**Figure 5:**
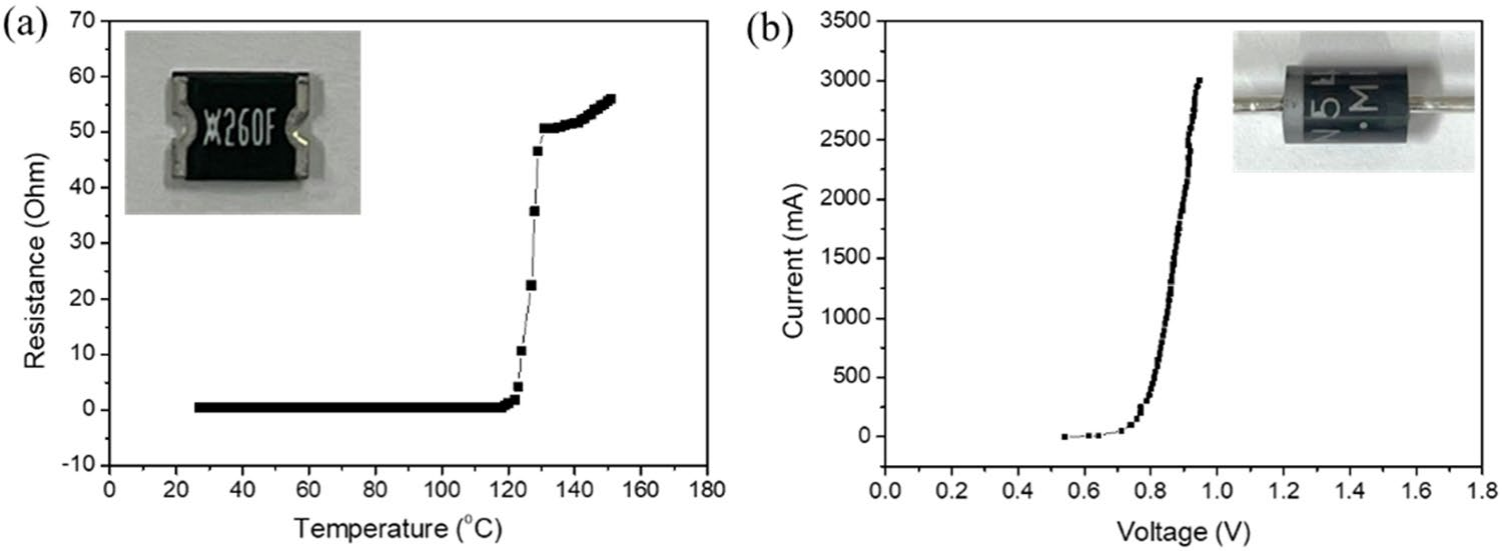
(**a**) Temperature dependency for positive thermal coefficient (PTC) thermistor’s resistance (**b**) I–V characteristic of IN5408 diode.

**Figure 6:**
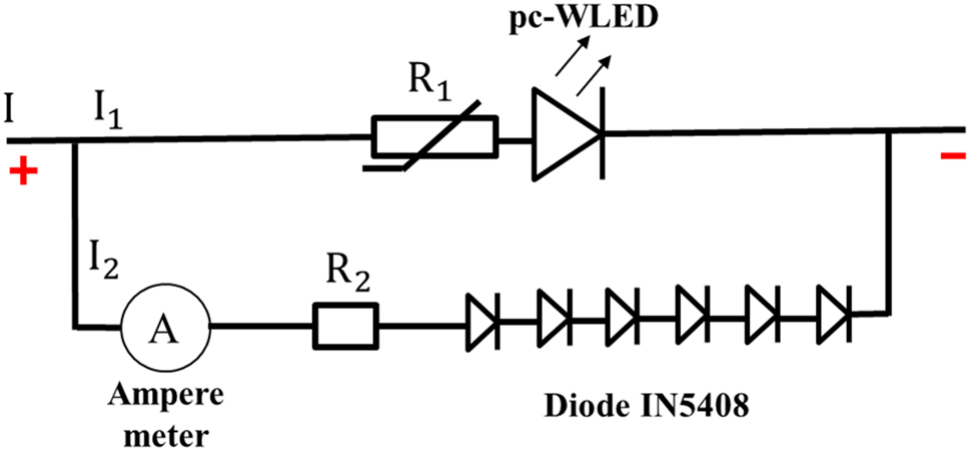
Circuit design for preventing blue light leakage.

As bluish light is not easily sensed once it occurs, we based our design on the relationship between the temperature characteristic and the blue leakage condition. We used a thermal sensor, i.e., a positive thermal coefficient (PTC) thermistor, to sense the overheat condition. When the overheat appeared, the PTC thermistor reduced the current flowing through the pcW-LEDs, thereby causing the flux quenching for the pcW-LEDs. The reduced amount of electrical current for the pcW-LEDs was redirected to a second branch including non-radiative diodes and a fixed resistor. The temperature dependency of the PTC is shown in Fig. 5a. The I–V characteristic of the non-radiative diode is shown in Fig. 5b. A diagram of the circuit connections is shown in Fig. 6.

The circuit connections can be described as follows. The upper branch includes the PTC thermistor and pcW-LEDs connected in series. The lower branch includes the fixed resistor and a series of six nonradiative diodes (type IN5408). The IN5408 diodes were selected owing to their ability to allow a large electrical current to flow through, i.e., up to 3 A. The upper branch and lower branch are connected in parallel. The circuit is powered by a constant electrical current.

With the above description, the relation between electrical current and voltage in the circuit can be expressed as follows:

1 $$I = I_{1} + I_{2}$$

and

2 $$I_{1} R_{1} + V_{LED} = I_{2} R_{2} + V_{D}$$

where I, I_1_, and I_2_ are the electrical currents in the whole circuit, upper, and lower branches, respectively. R_1_ is the resistance of the thermistor, and is temperature-dependent; R_2_ is the total resistance of the fixed resistor; V_D_ is the notation for the total drop voltage of the six IN5408 diodes, and V_LED_ is the voltage of the pcW-LEDs. Based on the relationships between the quantities in Eqs. (1) and (2), we can deduce the relationship as follows:

3 $$I_{1} = \frac{{V_{D} - V_{LED} + IR_{2} }}{{R_{1} + R_{2} }}$$

and

4 $$I_{2} = \frac{{V_{LED} - V_{D} + IR_{1} }}{{R_{1} + R_{2} }}$$

**Figure 7:**
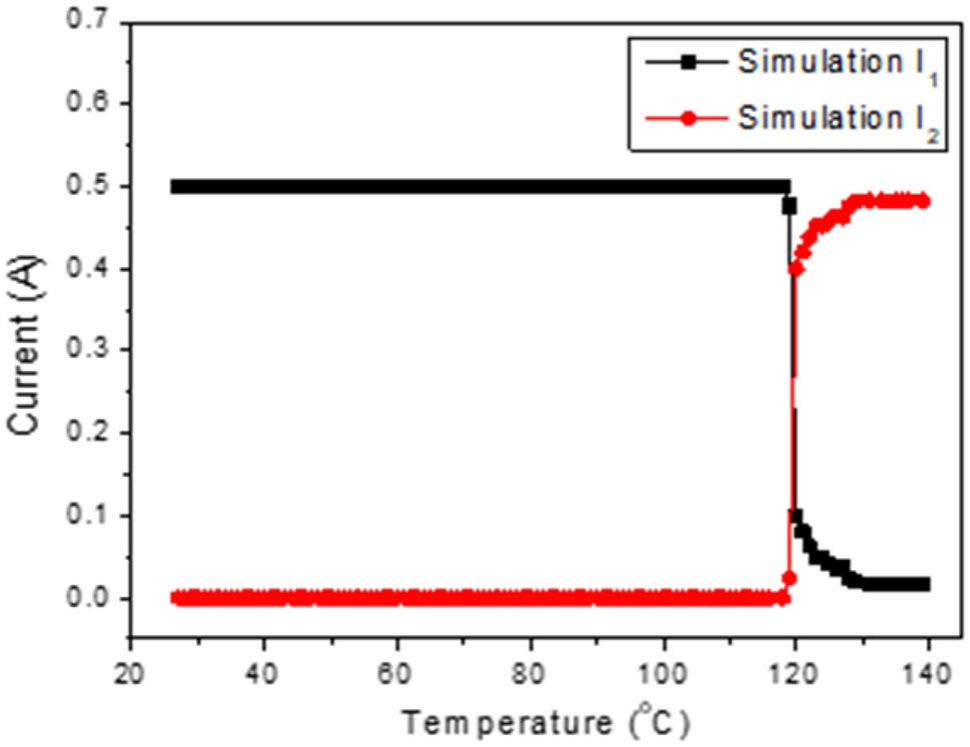
Changing of current in circuit versus temperature in simulation.

As the necessary parameters are known, the changing of the currents I_1_ and I_2_ in each branch corresponds to the working of the PTC thermistor in the normal condition (without overheating happening) and abnormal conditions (with overheating happening), making this approach suitable for simulation. In detail, the parameters used in the simulation included constant and variable parameters described as follows. The constant parameters included the voltage of the pcW-LED (V_LED_), resistance of the fixed resistor (R_2_), and total injection current (I), injected for the entire circuit. The value of V_LED_ used in the simulation was 3.1 V. It was assumed that the temperature dependence of V_LED_ was neglectable. The value of R_2_ used in the simulation was 1.3 Ω. The total injection current I for the entire circuit used in the simulation was 0.5 A. The variable parameters included the resistance of the thermistor (R_1_) and the total drop voltage of the six diodes (V_D_). The value of R_1_ was obtained from the resistance–temperature-dependent curve shown in Fig. 5a. In the circuit connection, the six IN5408 diodes were connected in series. The value of each diode is used from the I–V curve of the IN5408 diode, as is shown in Fig. 5b. The corresponding result from the simulation is shown in Fig. 7.

The working principle of the circuit for preventing the blue light leakage can be explained as follows. In normal conditions, there is no overheating; thus, the temperatures in the circuit and region where the PTC thermistor is connected are less than 120 °C. The PTC thermistor detects this temperature state and only works in the low-resistance mode. It thus has no effect on the electrical current in the circuit. Accordingly, the electrical current is only flowing in the upper branch, and there is no electrical current flow in the lower branch. In abnormal conditions, the overheating raises the temperature in the circuit and in the region where the PTC thermistor is connected to over 120 °C. The PTC thermistor detects the signal of the overheating state and rapidly activates the high-resistance mode. The resistance of the PTC thermistor is increased exponentially to cause a larger obstruction for the current as passing the upper branch. As a result, the current flow in the lower branches (I_2_) increases quickly while the current in the upper branches (I_1_) decreases correspondingly. Therefore, the injection current for the pcW-LED is quickly reduced, and the output flux of the light is significantly suppressed.

## Experimental measurement

**Figure 8:**
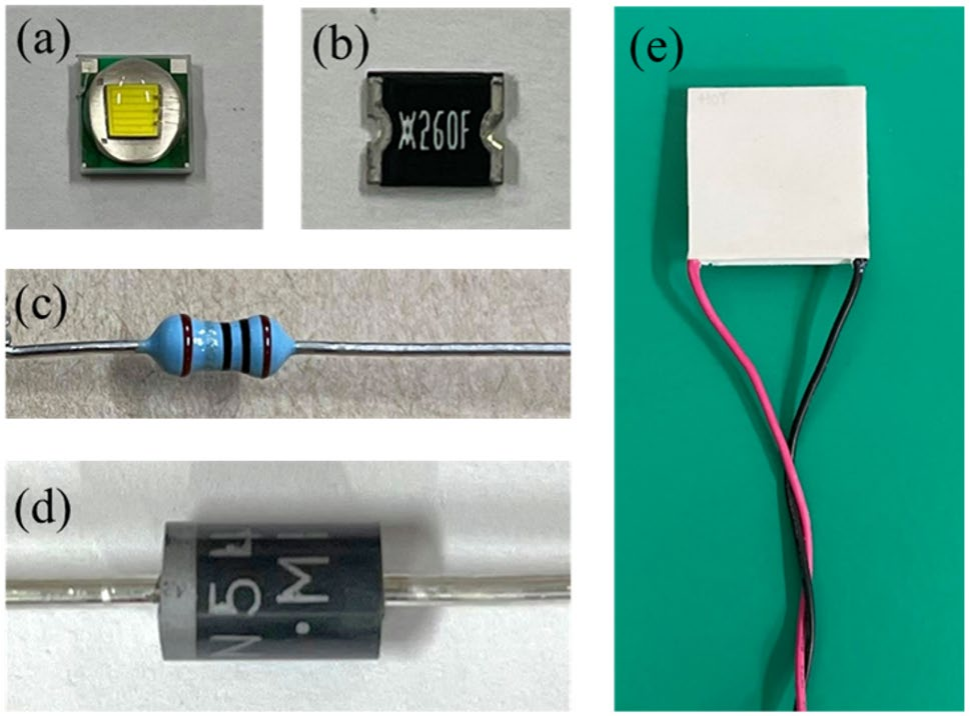
Components used in experiment, (**a**) pc-WLEDs, (**b**) PTC thermistor, (**c**) fixed resistor, (**d**) diodes, (**e**) thermoelectric cooler (TEC).

**Figure 9:**
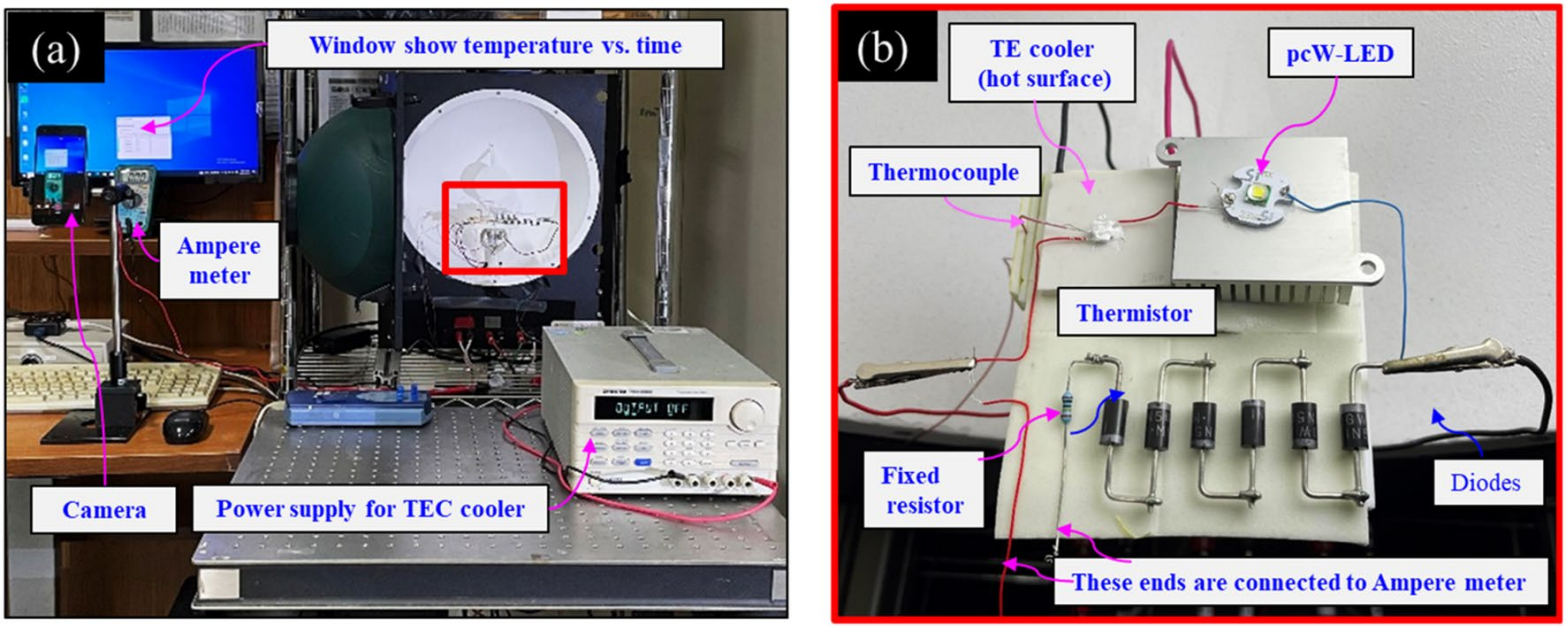
(**a**) Experiment set up for testing the changing of current with different detected temperatures by PTC thermistor. (**b**) Enlarged part of the red marked rectangle in the photo (**a**).

To confirm the changing of the electrical current from the simulation, a corresponding experiment was conducted. Figure 8 shows the components used in the experiment, including the pcW-LEDs, PTC thermistor, fixed resistor, nonradiative diodes, and thermoelectric cooler (TEC), respectively. The corresponding connection diagram for the experiment is similar to that shown in Fig. 6, but the TEC is added to heat the PTC thermistor. The experimental setup for testing the changing of the current with different temperatures being detected by the PTC thermistor is shown in Fig. 9. To cause the change in the resistance of the PTC thermistor, the thermistor is heated by the hot surface of TEC component. The temperature is detected by the thermocouple (type T) located very close to the interface of the TEC hot surface and PTC thermistor. A thermal conductivity gel with thermal conductivity of 1.8 W/m K is used to provide good thermal conduction. The temperature value is detected in real-time by using a PicoLog TC08 instrument connected to a computer. The electrical current injected for the whole circuit (I) is measured based on long-term testing of the integrating sphere system. The electrical current in the lower branch (I_2_) is measured by an amperemeter. A camera is used to record the computer desktop and the amperemeter screen simultaneously, thereby showing the changing temperature and electrical current values over time.

**Figure 10:**
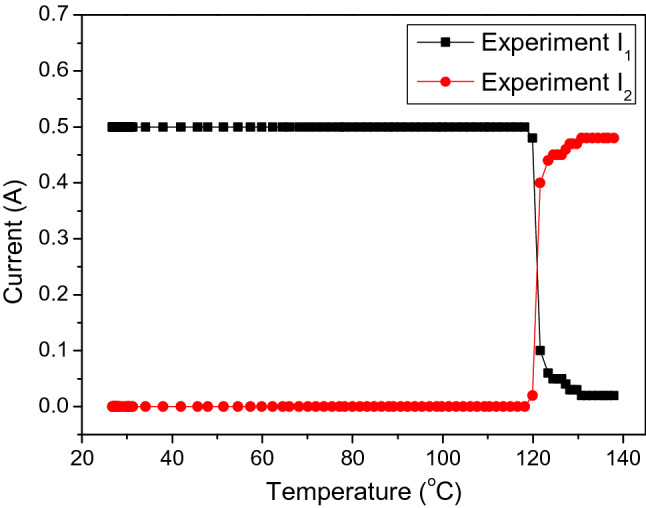
Changing of current in circuit versus temperature in the experiment.

The experimental results from changing the electrical current in each branch of the circuit are shown in Fig. 10. When the temperature is less than 120 °C, there is no change in the electrical current in each branch, because the PTC thermistor works in the low-resistance mode. When the temperature exceeds 120 °C, the PTC thermistor is activated to work in the high-resistance mode, causing a larger obstruction for the current as passing through it. The electrical current in the upper branch (I_1_) is therefore redirected to the lower branch, causing the reduction of I_1_ in the upper branch. The reduced I_1_ contributes to the increase of the electrical current in the lower branch (I_2_). It is necessary to compare the temperature dependence of the electrical current in the circuit between the simulation results and experimental results. Based on the qualitative comparison between the results shown in Figs. 7 and 10, the experiment and simulation results show high similarity.

## Demonstration of the designed circuit

**Figure 11:**
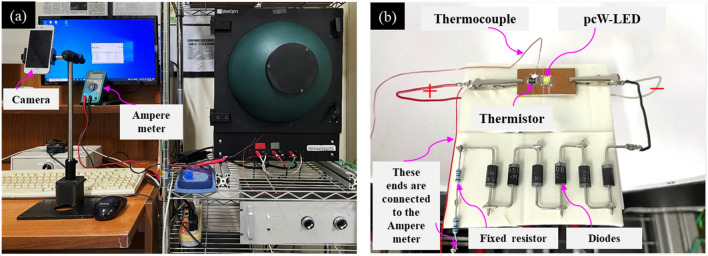
(**a**) Experimental set up. (**b**) The structure is set up inside the integrating sphere.

The ultimate task is the confirmation of the flux quenching owing to the current reduction when overheating occurs in the real circuit connection. Therefore, a real circuit was fabricated, and a corresponding experiment was performed. The experimental setup and real circuit (the latter being placed inside an integrating sphere) are shown in Fig. 11a and b, respectively. In this experiment, the overheating was generated by overdriving rather than by being heated up by the hot surface of TEC device. The temperature was measured in real-time by a thermocouple connected to a PicoLog TC08 instrument. The optical properties were measured using the long-term test mode of the integrating sphere system. The electrical current injection for the entire circuit (I) was set based on a constant current mode and could be detected in real-time and extracted from the results from the long-term test mode of the integrating sphere. The value of the electrical current in the branch containing the fixed resistor and nonradiative diodes (I_2_) was detected by an amperemeter. A camera was set up to record the changing of the electrical current I_2_ over time, as well as the temperature values shown on the amperemeter and computer screen.

**Figure 12:**
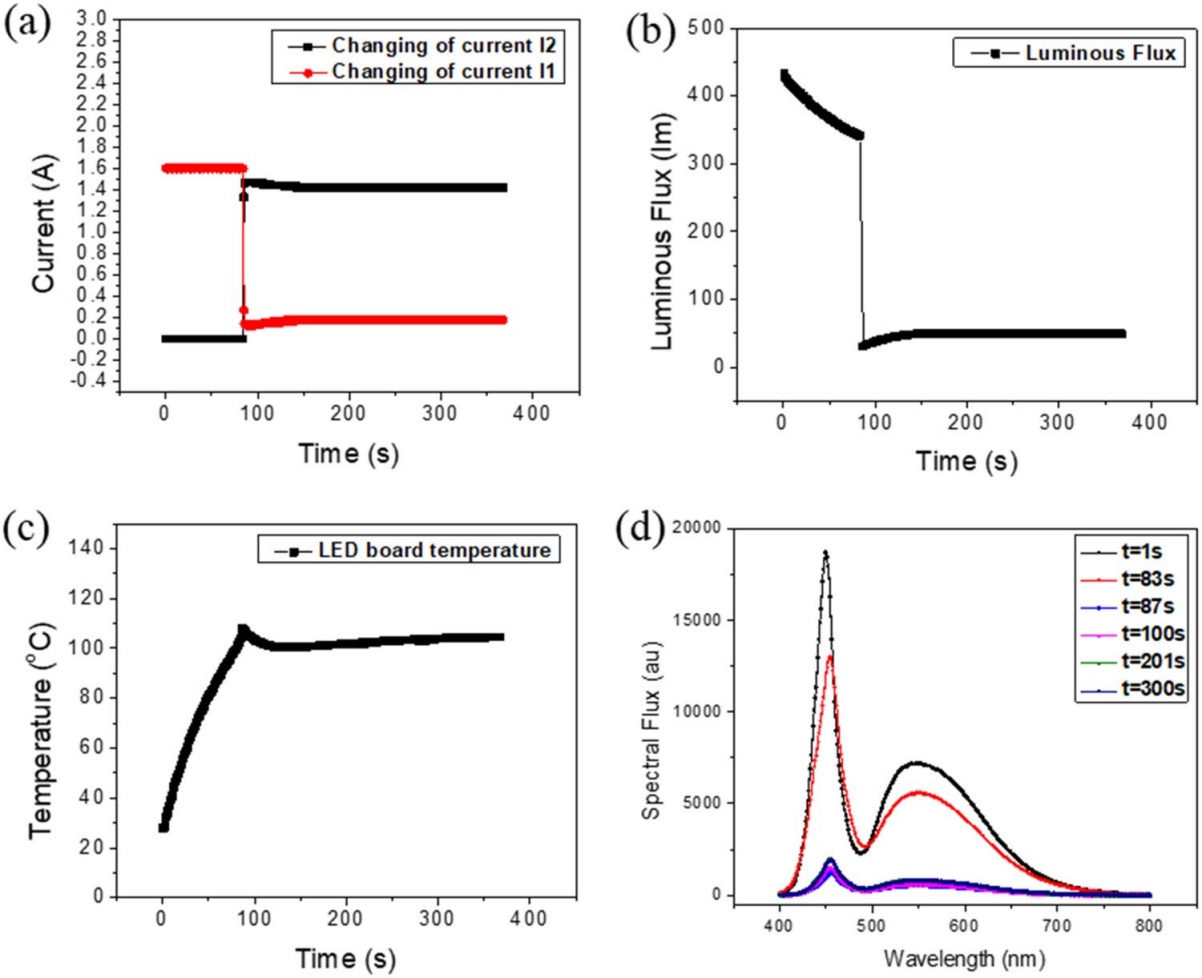
(**a**) Changing of electrical current. (**b**) Quenching of output flux. (**c**) The temperature variation. (**d**) Spectra of output light before/after the current injected to pcW-LEDs is reduced.

The results from the experiment are shown in Fig. 12. When serious overheating occurs, the temperature of the LED board increases quickly, and the PTC thermistor heats up. When the temperature of the PTC thermistor exceeds the working temperature of the PTC thermistor, the high-resistance mode is activated and reduces the electrical current in the circuit branch containing the pcW-LEDs (I_1_), as shown in Fig. 12a. Simultaneously, the reduced amount of current (I_1_) being transferred in the branch containing the fixed resistor and nonradiative diodes makes the current I_2_ increase, as shown in Fig. 12a. Corresponding to the reduction of the current I1, the luminous flux is significantly and rapidly suppressed, as shown in Fig. 12b. When the overheating is well-controlled, the temperature of the LED board no longer increases, as shown in Fig. 12c.

To more clearly illustrate the flux quenching in terms of the spectrum, the spectra of the output light before and after the current is injected into pcW-LEDs are shown in Fig. 12d. The time points at 1 s and 83 s correspond to the times when the PTC thermistor is working in the low-resistance mode and the current I_1_ is not reduced. The spectrum at 83 s is lower than that at 1 s owing to the thermal quenching for the blue and yellow light. The spectrum at 87 s shows a significant suppression relative to that at 83 s. This quenching of the output spectrum is owing to the significant decrease of the current I_1_, as shown in Fig. 12a. The spectra at 100 s, 201 s, and 300 s, respectively, almost overlap with that at the time of 87 s, owing to the PTC thermistor continuing to work in the high-resistance mode; thus, I_1_ remains small. This indicates that the PTC works well to maintain the condition of the flux quenching.

**Figure 13:**
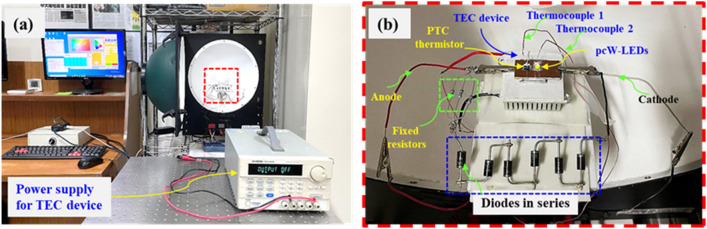
(**a**) Experimental setup for testing working of the circuit at normal, overheating, and after removing overheating conditions. (**b**) Enlarging of rectangle red dashed line in photo (**a**).

**Figure 14:**
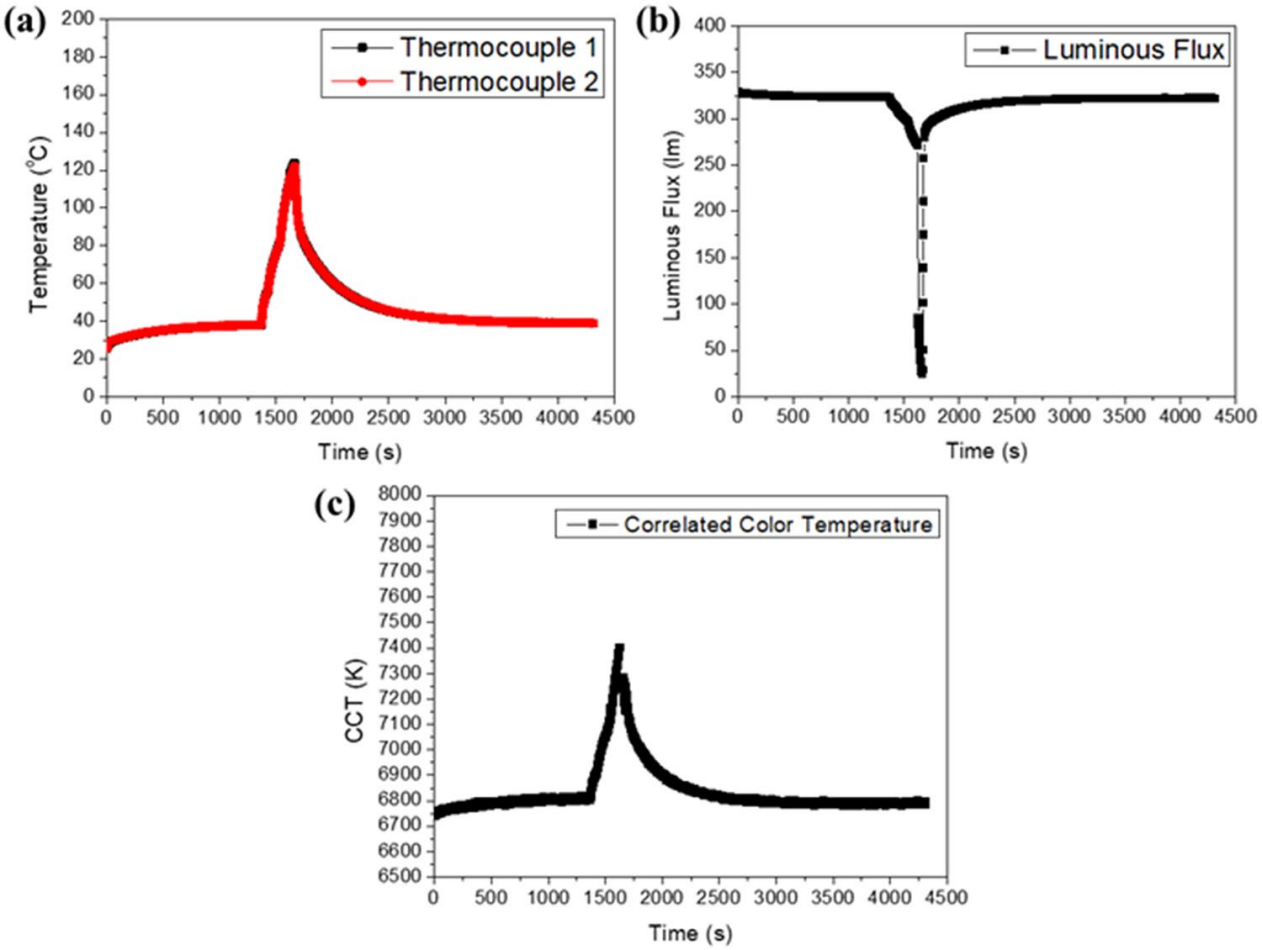
(**a**) Temperature, (**b**) Luminous flux, and (**c**) CCT behavior for three stages of normal, overheating, and after removing overheating conditions.

To further ensure the working of the design circuit in case of temperature is reduced after overheating condition is removed. Figure 13 shows the setup of this experiment which is similar to the setup in Fig. 11. A thermoelectric (TEC) device's hot surface is used to control the overheating condition for the circuit board containing the PTC thermistor and pcW-LEDs. The TEC device is operated by manually switching ON/OFF a power supply outside. The temperature is recorded by thermocouples 1 and 2, which are located close to the PTC thermistor and pcW-LEDs. Figure 14 shows the experimental results corresponding to three stages which are normal, overheating, and after removing overheating stage. Results show that, at the time of 1370 s, the overheating condition is generated by turning on the TEC, causing the temperature to be raised higher than the threshold to activate PTC thermistor to work in high resistant mode. The electrical current is prevented from flowing through the thermistor to inject into pc-WLEDs which suppresses output luminous flux as shown in Fig. 14b. At the time of 1670 s, the TEC device is turned off, the temperature is reduced to a lower value, and the PTC thermistor will return in the low resistant mode that leading to the increase for electrical current to pass through it and injecting for pc-WLEDs. As a result, it will see the recovery of output flux. In Fig. 14c, the CCT is raised due to the increase in temperature and is reduced to a lower value when overheating condition is removed. In summary, thanks to the particular characteristics of the PTC thermistor, the designed circuit can work well at different stages of temperature happen in the circuit.

## Conclusions

The unavoidable phenomenon of blue light leakage in pcW-LEDs has a strong relation to the appearance of an overheating condition. To our best knowledge, this is the first time a solution has been proposed and demonstrated successfully for the prevention of blue light leakage for pcW-LEDs driven by a constant electrical current power supply. A circuit was designed wherein the PTC thermistor was used as a thermal sensor for detecting overheating (which can generate the blue light leakage condition). When an overheating signal appears, blue light leakage can be effectively prevented based on activating a binary working mode corresponding to the PTC thermistor (corresponding to the normal and overheating conditions). After being activated, the low-resistance mode changes to the high-resistance mode, leading to a quick reduction of the electrical current injected to the LEDs. Therefore, the corresponding output luminous flux can be significantly suppressed, preventing the user's eyes from being exposed to bluish light. As a result, the lamp becomes dimmer. Furthermore, the dimmer state of the lamp also reminds the user that the lamp with the blue light leakage should be replaced. The solution is not only meaningful for the biological safety of humans, but also for increasing the lighting quality using pcW-LEDs in the field of SSL. Besides the advantages of its low cost, simplicity, and effectiveness, this circuit can easily be integrated into LED components owing to its simplicity.

## Data Availability

All data generated or analyzed during this study are included in this published article.
